# P38 MAPK signaling pathway mediates COM crystal-induced crystal adhesion change in rat renal tubular epithelial cells

**DOI:** 10.1007/s00240-019-01143-z

**Published:** 2019-06-10

**Authors:** Shiyong Qi, Qi Wang, Bin Xie, Yue Chen, Zhihong Zhang, Yong Xu

**Affiliations:** 1 Department of Urology, Tianjin Institute of Urology, The Second Hospital of Tianjin Medical University, 23 Pingjiang Road, Hexi District, Tianjin, 300211 China; 2 Department of Surgery, Linyi People’s Hospital, Linyi, Shandong China

**Keywords:** P38 MAPK, COM crystals, NLRP3 inflammasome, Calcium oxalate stone, NRK-52E

## Abstract

The objective of the study is to clarify the mechanism of p38 mitogen-activated protein kinase (p38 MAPK) signaling pathway in the change of crystal adhesion in rat renal tubular epithelial cells (NRK-52E) induced by calcium oxalate monohydrate (COM) crystals. NRK-52E cells were divided into COM crystal-treated group and control group according to whether the cell culture medium contains different concentrations of COM crystals. The concentrations of lactate dehydrogenase in the both group medium were determined after being cultured for 24 h. Protein and RNA were extracted from both cell groups after being cultured at different time points. SB239063, an inhibitor of the activation of p38 MAPK, was pretreated for 2 h before incubation with COM crystals. Western blotting and RT-qPCR were performed to confirm the expression levels of relative genes. All the experimental results were summarized and analyzed by SPSS 20.0 statistical analysis software. COM crystals (146 µg/cm^2^) could induce the expression levels of NLRP3, caspase-1 and interleukin-1β (IL-1β) significantly increased in NRK-52E cells. Compared with the control group cells, the transcription and translation levels of p38 MAPK-related molecule (such as p-p38) and adhesion molecules (such as osteopontin, hyaluronic acid and CD44) were significantly increased in COM crystal-treated cells and can be inhibited by SB239063 and NLRP3 gene silencing. This study demonstrated that the p38 MAPK signaling pathway mediated the COM crystal-induced crystal adhesion change in NRK-52E cells and required the involvement of NLRP3 inflammasome.

**Electronic supplementary material:**

The online version of this article (10.1007/s00240-019-01143-z) contains supplementary material, which is available to authorized users.

## Introduction

Renal stones are one of the most common diseases in urology; about 5% of American males will be involved in their lifetime, and the percentage is still increasing [1]. Calcium oxalate is one of the most common components of kidney stones and involved in the formation of 80% renal stones [2]. Studies had demonstrated that the damage of renal tubular epithelial and the change of crystal adhesion in renal tubular epithelial cells were the key chains in the formation of calcium oxalate stones [3, 4]. However, the underlying mechanisms participating in the change of crystal adhesion in renal tubular epithelial cells were still unclear.

The nucleotide-binding oligomerization domain-like receptor protein 3 (NLRP3) inflammasome is a well-known cytoplasmic protein complex that associated with various metabolic diseases [5–7]. Knauf et al. found that, compared with wild-type mice, the incidence of oxalate nephropathy and renal failure was significantly reduced in NLRP3^−/−^ mice that fed with the diet high in soluble oxalate [8]. Mulay et al. demonstrated that renal inflammation induced by calcium oxalate crystals was mediated by NLRP3 inflammasome [9]. Joshi et al. found that the deposition of calcium oxalate crystals and the expression of NLRP3 in renal tissues were elevated in kidneys of rats fed with hydroxy-l-proline (HLP) [10]. However, the role of the NLRP3 inflammasome in the adhesion of COM crystals in the renal tubular epithelium was still unclear and need further researches to verify.

The adhesion of COM crystals to renal tubular epithelial cells is the basis for the onset of calcium oxalate stone disease. Former studies had illustrated that hyaluronic acid (HA) was a high molecular mass polysaccharide and up-regulated in kidney tissue during inflammation [11, 12]. Hyaluronan synthases (HAS) was a key enzyme for HA synthesis and had three isoenzymes, termed as HAS1, HAS2 and HAS3, respectively [13]. Osteopontin (OPN) was a kind of glycoprotein and was significantly up-regulated during inflammation [13]. Studies had demonstrated that CD44 was a cell surface receptor for HA and OPN and also up-regulated during renal inflammation [13, 14]. Marino Asselman et al. demonstrated that the retention of calcium oxalate crystals required renal tubular epithelial damage and the expression of HA, OPN and CD44 in the rat kidneys [15].

P38 MAPK, one of the isozymes of MAPK, participated in the onset of atherosclerosis by directly affecting the expression of collagen [16]. The study of Paleerath et al. illustrated that the disruption of tight junction in epithelial cells was mediated by p38 MAPK signaling pathway and the expression level of related proteins of p38 MAPK signaling pathway were up-regulated during calcium oxalate stone formation [17]. On these bases, we could hypothesize that the COM crystal-induced over-expression of adhesion molecules are mediated by p38 MAPK signaling pathway and NLRP3 inflammasome plays an important role in it.

## Materials and methods

### Preparation of COM crystals

The preparation of COM crystals was referenced from the study of Thongboonkerd et al. [18]. Calcium chloride dihydrate (10 mM) was mixed with sodium oxalate (10 mM), and a mixture of a final concentration of 5 mM and 0.5 mM was prepared in Tris buffer containing 90 mM NaCl. The mixture was incubated overnight at 25 °C and COM crystals were harvested by centrifugation at 3000 rpm for 5 min. The supernatant was discarded and the crystals were re-suspended in methanol. After further centrifugation at 3000 rpm for 5 min, methanol was discarded and the crystals were dried overnight at 37 °C. Then the COM crystals were decontaminated by UV light for 30 min. Finally, they were added to complete Eagle's minimum essential medium (MEM) to reach a final concentration of 1000 µg/ml*.*

### Cell culture

The rat kidney proximal tubular epithelial (NRK-52E) cells were purchased from SIBS (Shanghai Institutes for Biological Sciences). NRK-52E cells were cultured in high-glucose DMEM supplemented with 10% fetal bovine serum (FBS) and 1% penicillin/streptomycin (P/S) under 5% CO_2_/95% atmosphere at 37 °C incubator, and serial sub-cultivation in T-25 flasks. DMEM, FBS, penicillin and streptomycin were bought from Gibco (USA), NRK-52E cells were cultured in 6- and 24-well plates. COM crystals were added at final different concentrations of 0, 36.5, 73.0, 109.5, 146, and 182.5 µg/cm^2^, respectively. NRK-52E cells grew to 70–80% confluence in complete medium and the different concentrations of COM crystals were added, after incubated for 0, 12 and 24 h, the cells and medium were separately collected for further analysis. SB239063 (purchased from Sigma), an inhibitor of the activation of p38 MAPK, was prepared in PBS. SB239063 was added at final different concentrations of 20 mM and incubated with NRK-52E cells for 2 h and then the cells were collected for further analysis.

### LDH release

LDH released into cell culture medium was induced by cell membrane injury. NRK-52E cells were seeded in six-well plate as mentioned above and COM crystals were added at final different concentrations of 0, 36.5, 73.0, 109.5, 146.0, 182.5 µg/cm^2^. After incubating with cells for 24 h, LDH was measured by LDH-kit (Nanjing Jiancheng Bioengineering Institute) as per the constructor’s protocol. The final result was measured by Microplate Reader at 450 nm.

### Immunofluorescence staining

NRK-52E cells were seeded in 24-well plates via the method of coverslip culture, while NRK-52E cells grew to 30–50% confluence in complete medium, COM crystals were added at final concentration of 146.0 µg/cm^2^ and incubated for 24 h. After incubation overnight with primary antibodies anti-NLRP3 (1:50, Abcam, Cambridge, UK) and anti-caspase-1 (1:50, Abcam) and then incubating with secondary antibodies, images were obtained with the fluorescence microscope (Olympus, Tokyo, Japan).

### Small interfering RNA (siRNA) knockdown experiments

Double-stranded siRNA targeting NLRP3 gene and a negative control (NC) siRNA were purchased from GenePharma (Shanghai, China). NRK-52E cells were transfected using siRNA mixed with Lipofectamine 2000 (Invitrogen, USA) following the manufacturer’s protocol. The NLRP3-siRNA sequence is 5′-GGAGAGACCUUUAUGAGAATT-3′ and 5′-UUCUYCAUAAGGUCUCUCCTG-3′ as a reverse sequence. Negative control sequence is 5′-UUCUCCGAACGUGUCACGUTT-3′ and the reverse sequence is 5′-ACGUGACACGUUCGGAGAATT-3’.

### Western blotting analysis

Protein extractives were lysed in mammalian cell lysis buffer. Protein concentration was determined referring to a bovine serum albumin standard. Proteins were separated on 10–12% SDS-PAGE gels. Protein expression levels were determined using primary antibodies anti-NLRP3 (1:500, ab214185), anti-caspase-1 (1:500, ab108362), anti-IL-1β (1:500, ab2105), anti-HAS1 (1:500, ab198846), anti-p-p38 (1:500, ab45381) and anti-p38 (1:500, ab31828), which were purchased from Abcam (Cambridge, UK), and primary antibodies anti-CD44 (1:1000, 15675–1-AP), anti-OPN (1:1000, 22952–1-AP) were purchased from Proteintech (USA). Anti-rabbit and anti-mouse IgG secondary antibodies were purchased from Santa Cruz (USA). GAPDH (Santa Cruz, USA) was used as loading control.

### Scanning electron microscopy (SEM)

Experimental NER-52E cells were seeded in 24-well plates via the method of coverslip culture. The cells were treated with or without COM crystals for about 24 h and COM crystal exposure cells were also divided into two groups according to whether pretreatment with SB239063 at 20 mM for 2 h, and then all cells were fixed with glutaraldehyde at 4 °C refrigerator through the night. After that, the cells were viewed and imaged using a Hitachi S-3400 N SEM.

### Real-time quantitative polymerase chain reaction (RT-qPCR) analysis

The total RNA from NRK-52E cells were extracted using the Trizol Reagent (Invitrogen, USA), and cDNA was then synthesized using a reverse transcription (RT) system kit (Thermo Fisher Scientific, USA) according to the manufacturer’s instructions. RT-qPCR was performed using the SYBR Premix Ex Taq II (Takara Biotechnology, China) following the manufacturer’s protocols. The sequences of related primers used in this study are shown in Table 1. The amplification and analysis were performed on a Real-Time PCR system (ABI prism 7500, Applied Biosystems, USA). All of the data were analyzed with − ΔΔ*C*_*t*_ method and normalized using GAPDH cDNA as an internal control.

**Table 1:** Sequences of related primers for research

	Forward	Reverse
NLRP3	5′-CGTGAGTCCCATTAAGATGGAGT-3′	5′-CCCGACAGTGGATATAGAACAGA-3′
P38	5′-TTTCCGCAAGGTTCGATTTTCA-3′	5′-GGCATCTGCGCTCTACCATC-3′
HAS1	5′-CACTGTGTATCCTGCATCAG-3′	5′-CTTGGTAGCATAACCCATGC-3′
HAS2	5′-CTCAGTGTTATACATGTCGAGTTTACTTC-3′	5′-ACTGATACTGGAATGAGTCCTATGAA-3′
HAS3	5′-AGTGCAGCTTCGGGGATGA-3′	5′-TGATGGTAGCAATGGCAAAGAT-3′
QPN	5′-AGCACAGCATCGTCGGGAC-3′	5′-TCCTTGGTCGGCGTTTGGCTG-3′
CD44	5′-CTGTCTGTGCTGTCGGTGAT-3′	5′-CATCTACCCCAGCAACCCTA-3′

## Results

### COM crystals could induce the formation of NLRP3 inflammasome

**Fig. 1:**
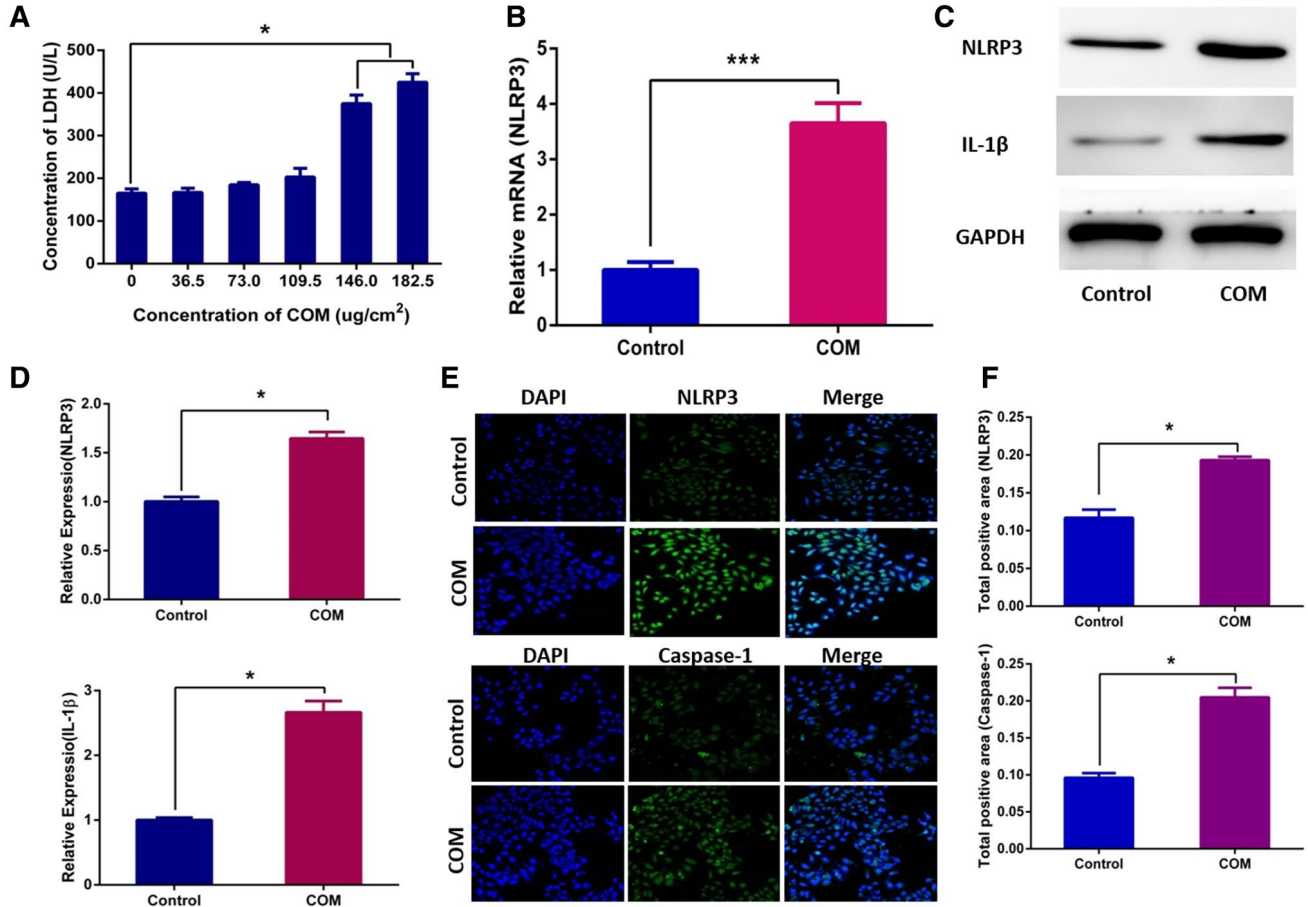
COM crystal could induce the formation of NLRP3 inflammasome. **a** Detection of the concentrations of LDH in NRK-52E cells culture medium performed after NRK-52E cells were incubated with differential concentration of COM crystals for 24 h. *P < 0.05; **b** RT-qPCR was used to detect the expression level of NLRP3 mRNA in NRK-52E cells exposed with COM (146.0 µg/cm^2^), whereas GAPDH served as the loading control. ***P < 0.005; **c** Western blotting was used to measure the expression levels of NLRP3 and IL-1β in NRK-52E cells exposed to COM (146.0 µg/cm^2^), GAPDH served as the loading control; **d** band intensities of NLRP3 and IL-1β, respectively, were measured by a densitometer and normalized with that of GAPDH, *P < 0.05; **e** immunofluorescence staining analysis of the expression levels of NLRP3 (green) and caspase-1 (green) in NRK-52E cells exposed with COM (146.0 µg/cm^2^), the nucleuses were stained with DAPI (blue) and the magnification of all images is 200; **f** total positive area of NLRP3 and caspase-1 were measured by Image J software, *P < 0.05. *N* = 3 independent experiments for each bar

The intracellular lactate dehydrogenase (LDH) was released into the cell culture medium when cells were dead and we indirectly measured the effect of different concentrations of COM crystals on cell death by detecting the concentration of LDH in the cell culture medium. The concentration of LDH in culture medium was detected after NRK-52E cells were incubated with different concentrations (0, 36.5, 73.0, 109.5, 146.0, 182.5 µg/cm^2^) of COM crystals for 24 h. The results revealed that, compared with low concentrations (36.5, 73.0, 109.5 µg/cm^2^) of COM crystals, the cell death significantly increased when incubated with higher concentrations (146.0, 182.5 µg/cm^2^) of COM crystals (Fig. 1a). By measuring the expression level of NLRP3 in COM-treated NRK-52E cells, we found that COM crystals (146.0 µg/cm^2^) could induce the transcription and translation level of NLRP3 significantly increased (Fig. 1b, c, e). IL-1β has been illustrated to be activated by the activation of NLRP3 inflammasome [19] and we also found the expression level of it also significantly increased in COM crystal-treated cells (Fig. 1b). Procaspase-1, a component of NLRP3 inflammasome, can be cleaved into its active form caspase-1 by the formation of NLRP3 inflammasome [20]. Our study also demonstrated that COM crystals (146.0 µg/cm^2^) could induce the production of caspase-1 (Fig. 1e). From the above findings, we concluded that COM crystals could induce the formation of NLRP3 inflammasome in NRK-52E cells.

### SB239063 can inhibit the COM crystal-induced activation of p38 MAPK signaling pathway in NRK-52E cells

**Fig. 2:**
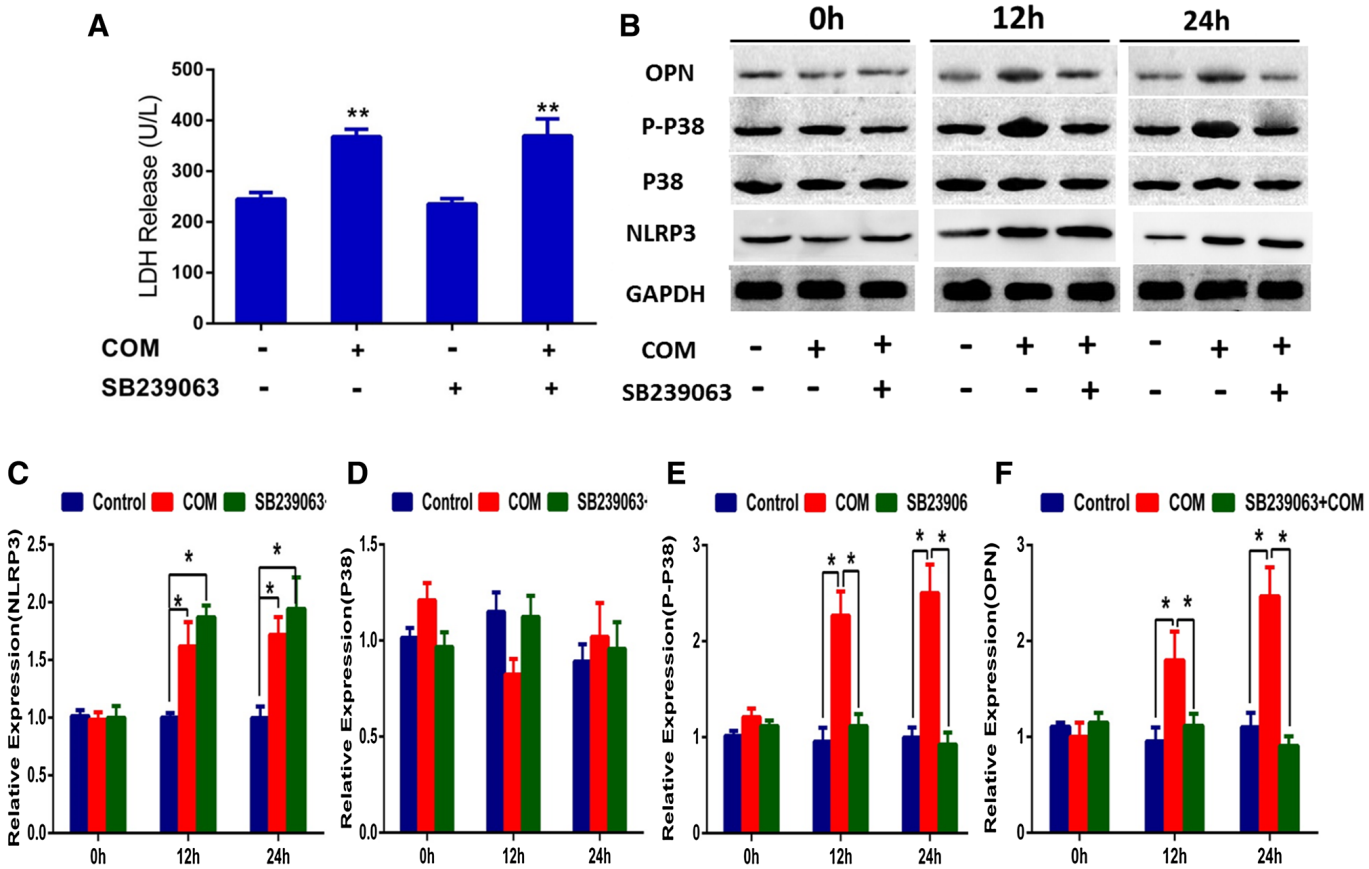
Expression levels of related proteins in SB239063-treated-NRK-52E cells. **a** The cell toxicity of SB239063 was detected by LDH-kit; **P < 0.01 VS. control group; **b** proteins derived from whole cell lysates of controlled and COM-treated (146.0 µg/cm^2^) cells at different time points (0, 12, 24 h), without or with 2-h pretreatment by 20 mM SB239063 (an inhibitor of p38MAPK activation), were equally loaded into each of SDS-PAGE lane. The proteins were subjected to Western blotting analysis including anti-NLRP3, anti-p38, anti-p-p38 and anti-OPN. GAPDH served as the loading control. **c**–**f** Band intensities of NLRP3, p38, p-p38 and OPN, respectively, were measured by a densitometer and normalized with GAPDH. *N* = 3 independent experiments for each bar; *P < 0.05

Phospho-p38 (p-p38), the active form of p38, was performed to examine the effects of COM crystals on p38 MAPK signaling pathway in NRK-52E cells. The expression level of total p-38 was directly detected by p-38. The data revealed that COM crystal exposure of NRK-52E cells had induced the expression level of p-p38 significantly increased. SB239063, a specific inhibitor of the activation of p38 MAPK signaling pathway, was non-toxic to NRK-52E cells (Fig. 2a), was treatment with cells for 2 h at 20 mM before the incubation of COM crystals. According to the results, we found that the expression level of p-p38 was significantly increased when incubated with COM crystals for 12 or 24 h, meanwhile the expression level of p38 has not changed (Fig. 2b). Thus, we concluded that COM crystals could activate p38 MAPK signaling pathway by inducing the phosphorylation of p38. At the same time, our study also found that SB239063 can effectively inhibit the COM crystal-induced up-regulation of the expression level of p-p38 (Fig. 2b). OPN, a kind of adhesion molecule, was up-regulated in NRK-52E cells when incubated with COM crystals for 12 or 24 h, and which also could be suppressed by SB239063 (Fig. 2b). Thus, we concluded that the COM crystal-induced change of crystal adhesion in NRK-52E cells was mediated by p38 MAPK signaling pathway and can be prevented by SB239063.

### NLRP3 gene silencing can prevent the COM crystal-induced overproduction of NLRP3, p-p38 and OPN in NRK-52E cells

**Fig. 3:**
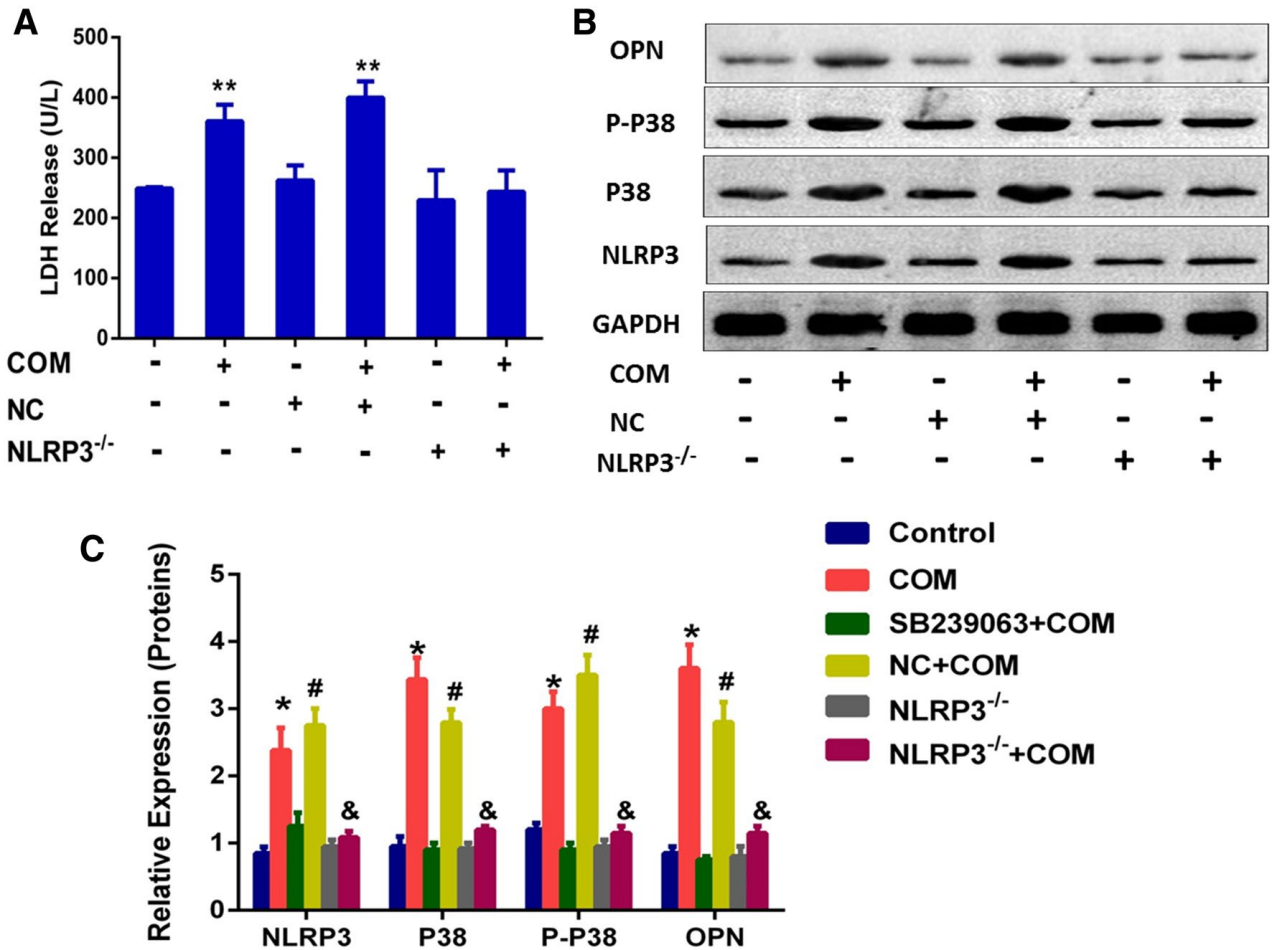
Expression levels of related proteins in NLRP3 gene-silenced NRK-52E cells. **a** The cell toxicity of NLRP3-siRNA was detected by LDH-kit; **P < 0.05 vs. control group; **b** proteins derived from whole cell lysates of controlled and COM-treated (146.0 µg/cm^2^) cells without or with transfection of NLRP3-SiRNA or NC, were equally loaded into each of SDS-PAGE lane. The resolved proteins were then subjected to Western blot analysis for NLRP3, p38, p-p38 and OPN, whereas GAPDH served as the loading control. **c** Band intensities of NLRP3, total p38, phospho-p38 and OPN, respectively, were measured by a densitometer and normalized with GAPDH. *N* = 3 independent experiments for each bar; *P < 0.05 vs. control group; ^#^*P* < 0.05 vs. NC group; **P < 0.05 vs. COM group or COM + NC group

The above experiments have demonstrated that the p38 MAPK signaling pathway plays an important role in the COM crystal-induced change of crystals adhesion in NRK-52 cells; however, the relationship between NLRP3 inflammasome and p38 MAPK signaling pathway was still unclear. First of all, we found that the cell viability of NRK-52E cells was not affected by LDH-kit (Fig. 3a). By detecting the transcription and translation levels of corresponding markers in NLRP3 gene-silenced NRK-52E cells, we found that NLRP3 gene silencing in NRK-52E cells could prevent the COM crystal-induced overproduction of NLRP3, p-p38 and OPN (Fig. 3b). These results suggested that the p38 MAPK signaling pathway-mediated change of crystal adhesion in NRK-52E cells needs the involvement of NLRP3 inflammasome and the p38 MAPK signaling pathway was the downstream of NLRP3 inflammasome.

### NLRP3 gene silencing or SB239063 could suppress the COM crystal-induced overproduction of adhesion molecules in NRK-52E cells

**Fig. 4:**
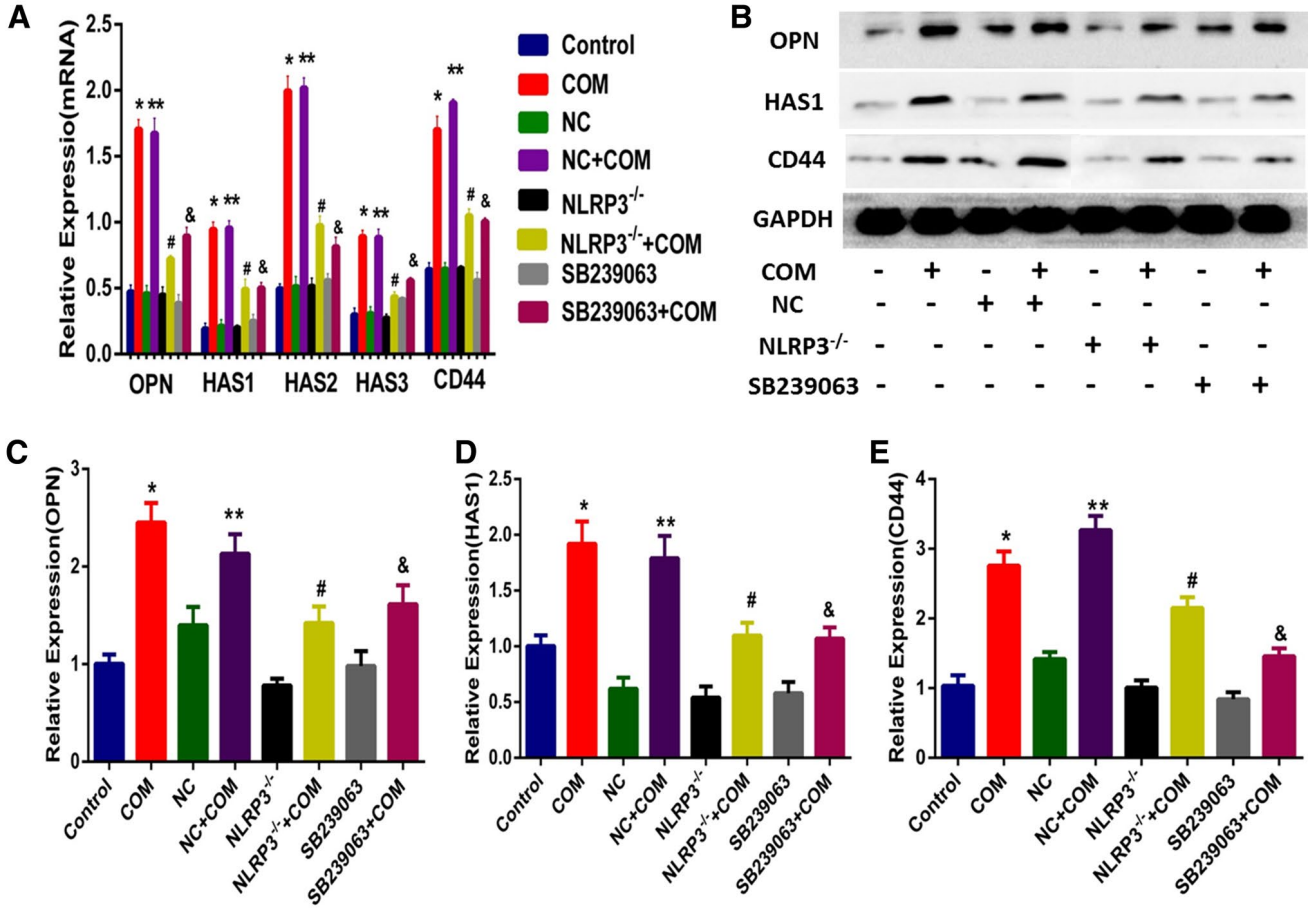
Detecting the expression levels of adhesion molecules. **a** Total RNA from whole cell lysates with different treatment, cDNA were synthesized according to the procedure of HiFiScript cDNA Synthesis Kit. RT-qPCR was used to detect the expression levels of HAS1, HAS2, HAS3, CD44 and OPN mRNA in NRK-52E cells exposed with COM (146.0 µg/cm^2^), whereas GAPDH served as the loading control. *N* = 3 independent experiments for each bar; *P < 0.05 vs. control; **P < 0.05 vs. NC group; ^#^*P* < 0.05 vs. COM group or COM + NC group; ^&^*P* < 0.05 vs. COM group or COM + NC; **b** Western blot analysis for CD44, HAS1 and OPN in NRK-52E cells exposed with COM (146.0 µg/cm^2^) and GAPDH served as the loading control. **c** Band intensities of CD44, HAS1 and OPN, respectively, were measured by a densitometer and normalized with GAPDH, *N* = 3 independent experiments for each bar; *P < 0.05 vs. control; **P < 0.05 vs. NC group; ^#^*P* < 0.05 vs. COM group or COM + NC group; ^&^*P* < 0.05 vs. COM group or COM + NC; **c**–**e** band intensities of OPN, HAS1, and CD44, respectively, were measured by a densitometer and normalized with GAPDH. *N* = 3 independent experiments for each bar; *P < 0.05 vs. control; **P < 0.05 vs. NC group; ^#^*P* < 0.05 vs. COM group or COM + NC group; ^&^*P* < 0.05 vs. COM group or COM + NC

Our study has demonstrated that COM crystals could induce the expression level of OPN up-regulated and which can be inhibited by NLRP3 gene silencing and SB239063. However, it is still unclear whether COM crystal treatment could increase the expression levels of other adhesion molecules, such as CD44 and HA. We indirectly reflect the expression level of HA by detecting the expression levels of HAS1, HAS2 and HAS3. Western blotting and RT-qPCR analysis were used to detect the expression levels of adhesion molecules in COM crystal-treated NRK-52E cells. The data revealed that the COM crystal exposure of NRK-52E cells could induce the transcription levels of HAS1, HAS2, HAS3, CD44 and OPN significantly increased, whereas SB239063 or NLRP3 gene silencing could prevent the COM crystal-induced overproduction of adhesion molecules on the surface of NRK-52E cells (Fig. 4a). Meanwhile, we also detected the translation levels of OPN, HAS1 and CD44 and found that the COM crystal-induced overexpression of OPN, HAS1 and CD44 in NRK-52E cells could be prevented by NLRP3 gene silencing and SB239063 (Fig. 4b). Thus, we can make a conclusion that NLRP3 inflammasome and p38 MAPK signaling pathway involved in the COM crystal-induced overproduction of adhesion molecules in NRK-52E cells.

### Visualization of crystal adhesion change in NRK-52E cells

The mechanism of COM crystal-induced crystal adhesion change in NRK-52E cells has been preliminary demonstrated; however, the microscopic performance of the crystal adhesion change in NRK-52E cells was still unclear. Control group cells and COM-treated NRK-52E cells, with or without the pretreatment of SB239063, were observed by SEM. Compared with the control group cells, we found that the COM crystal-treated cells had obvious morphological changes which were characterized by cell edema and the COM crystal adhesion to the cell membrane significantly increased. Meanwhile, the COM crystal-treated cells pretreated with SB239063 did not show the above changes (Supplementary Fig. 1).

## Discussion

P38 MAPK, a 38KD protein composed of 360 amino acids, combined with extracellular-signal regulated kinase 1/2 (ERK1/2) and c-Jun N-terminal kinase (JNK) to form the three major branches of MAPK signaling system [21]. P38 MAPK signaling pathway, composed of MAPK kinase kinase, MAPK kinase and p38 MAPK, was an important member of the MAPK family. Under the stimulation of various factors, such as hyperglycemia, pro-inflammatory factors, oxidative stress, the phosphorylation of MAPK kinase as followed by the phosphorylation of MAPK kinase and finally activated the p38 MAPK by inducing the phosphorylation of p38 MAPK residues [22]. The activated p38 MAPK signaling pathway participate in the processes of cell growth, differentiation, apoptosis, environmental stress response and inflammatory response [23].

Several studies had demonstrated that oxalate selectively activated the p38 MAPK signaling pathway and the activation of p38 MAPK signaling pathway was required for the re-initiation of oxalate-induced DNA synthesis in renal epithelial cells [24, 25]. The study of Han et al. found that oxalate could inhibit the proliferation of renal proximal tubule cells via the p38 MAPK/JNK signaling pathway and which can be prevented by the inhibitor of p38 MAPK signaling pathway [26]. Recent study also illustrated that the p38 MAPK pathway plays an important role in regulating the nephrotoxicity of oxalate in human renal epithelial cells [27]. Meanwhile, the study of Ilbey et al. demonstrated that pyrrolidine dithiocarbamate (PDTC) could prevent the deposition of crystals in renal tubules by reducing the expression levels of oxidative stress, iNOS, NF-kappa B and p38 MAPK [28]. Based on the above results, we could suspect that the p38 MAPK signaling pathway plays an important role in COM crystal-induced change of crystals adhesion in NRK-52E cells.

NLRP3 inflammasome, a kind of protein complex existing in the cytoplasm, includes NLRP3 receptor, apoptosis-associated speck-like protein containing CARD (ASC) and procaspase-1 [8]. The formation of NLRP3 inflammasome will consequently induce procaspase-1 cleaved into its active form caspase-1 [20, 29]. IL-1β, a kind of interleukin, was involved in the onset and progression of inflammation [30]. Studies have demonstrated that the activation of NLRP3 inflammasome will induce pro-IL-1β cleaved into its mature form IL-1β [9, 19]. Previous study has indicated that calcium oxalate crystals could induce innate immunity mediated by NLRP3/ASC/caspase-1 axis and IL-1β in intrarenal mononuclear phagocytes [9]. Our study has demonstrated that COM crystals could induce the overexpression of NLRP3, caspase-1 and IL-1β, which indirectly reflects the formation of NLRP3 inflammasome.

It is well known that the enhancement of adhesion between COM crystals and renal tubular epithelial cells was one of the key points in the onset and progression of calcium oxalate stones. OPN, CD44 and HA were the most studied adhesion molecules. The study of Asselman et al. found the damage of tubular epithelial and the over-expression of HA, OPN and CD44 in rat kidney with oxalate crystal deposition [15]. Then the study of Tsuji et al. suggested that the inhibition of OPN expression in the renal of hyperoxaluric rats also inhibited the deposition of renal crystals [31]. These results indicated that the enhanced adhesion of calcium oxalate crystals to renal tubular epithelial cells was achieved by the crystal-induced over-expression of adhesion molecules. At the same time, study also demonstrated that the aldosterone-induced expression of OPN in vascular smooth muscle cells was mediated by mineralocorticoid receptor (ER) and the involved signaling cascades including ERK and p38 MAPK [32]. The study of Zuo et al. illustrated that the atorvastatin-induced expression of OPN could be prevented by the inhibition of the p38 MAPK signaling pathway [33]. Recent study suggested that ROS–Akt–p38 MAPK signaling pathway was activated in COM crystal-induced disruption of tight junction in Madin–Darby canine kidney (MDCK) cells [34]. These data confirmed the hypothesis that the COM crystal-induced activation of p38 MAPK signaling pathway was associated with the change of crystal adhesion in NRK-52E cells.

Our study demonstrated that COM crystals could induce the over-expression of OPN in NRK-52E cells and this result was consistent with the former studies. In our study, we also found that COM crystals could induce the expression level of p-p38 significantly which directly demonstrated the activation of p38 MAPK signaling pathway. However, the overexpression of OPN and p-p38 can be prevented by the activation inhibitor of p38 MAPK signaling pathway. These results were consistent with other studies and proved that the up-regulation of adhesion molecules in NRK-52E cells induced by COM crystals was mediated by p38 MAPK signaling pathway. Other studies also had proved that NLRP3 inflammasome plays an important role in the pathogenesis of oxalate-induced nephropathy [8–10]. Our study also illustrated that NLRP3 gene silencing could suppress the up-regulation of OPN and p-p38, which directly proved that the COM crystal-induced crystal adhesion change was mediated by p38 MAPK signaling pathway and p38 MAPK signaling pathway was the downstream of NLRP3 inflammasome. Our study first reported the intuitive morphological changes of crystal adhesion in NRK-52E cells by scanning electron microscopy, and found that the changes can be abrogated by the activation inhibitor of p38 MAPK signaling pathway.

Based on our research and previous reports, the underlying mechanism of crystal adhesion change in NRK-52E cells induced by COM crystals can be explained as follows. First, the COM crystal incubation with the cell culture medium could induce the up-regulation of NLRP3 inflammasome in NRK-52E cells and then NLRP3 inflammasome activated the p38 MAPK signaling pathway by inducing the phosphorylation of p38. Finally, high expression levels of adhesion molecules were mediated by p38 MAPK signaling pathway thereby changing the adhesion of COM crystals in NRK-52E cells. The enhancement of COM crystal adhesion in NRK-52E cells could lead to the increase of COM crystals adhering to the renal tubular epithelium. With large COM crystal particles being formed, the calcium oxalate stones will be formed eventually.

Our study found that the reductions of NLRP3 inflammasome and p38 MAPK signaling pathway productions in NRK-52E cells were associated with the reduction of COM crystals adhering to NRK-52E cells. Our study also illustrated that the enhancement of crystal adhesion to NRK-52E cells induced by COM crystals was mediated by NLRP3 inflammasome and p38 MAPK signaling pathway and the underlying mechanisms in the onset and progression of calcium oxalate stones were further demonstrated. However, our study is not devoid of limitations. First, the results of in vitro experiments lack the validation of animal experiments. Second, although it has been illustrated that the COM crystal-induced change of crystal adhesion in NRK-52E cells requires the involvement of NLRP3 inflammasome and p38 MAPK signaling pathway, it is still unclear whether other signaling molecules or signaling pathways were involved in this process. Finally, various factors were involved in the formation of calcium oxalate stones in human kidneys and our study only simulates the effect of COM crystal exposure on NRK-52E cells. Therefore, additional studies should be performed to verify the specific underlying mechanisms of the crystal adhesion change in NRK-52E cells.

In summary, our study points out the important roles of p38 MAPK signaling pathway and NLRP3 inflammasome in COM crystal-induced change of crystal adhesion in NRK-52E cells. Our research has preliminary proved the pathogenesis of calcium oxalate stone disease, which provides a direction for the following researches in specific underlying mechanisms and the new ideas for the prevention and treatment of calcium oxalate stone disease.

## Electronic supplementary material

Below is the link to the electronic supplementary material.

Supplementary Fig. 1 Results of scanning electron microscopy. Controlled and COM-treated (146.0 µg/cm^2^) NRK-52E cells at 24 h, without or with 2-h pretreatment by SB239063 at 20 mM, were observed the changes of cell surface adhesion by SEM. COM crystal-treated cells had obvious swelling (blue arrows) and COM crystals adhesion significantly increased (red arrows). Scale bar is 20 µm

## Abbreviations

p38 MAPK: p38 mitogen-activated protein kinase
COM: Calcium oxalate monohydrate
LDH: Lactate dehydrogenase
NLRP3: Nucleotide binding oligomerization domain-like receptor protein 3
HLP: Hydroxy-l-proline
HA: Hyaluronic acid
HAS: Hyaluronan synthases
OPN: Osteopontin
FBS: Fetal bovine serum
P/S: Penicillin/streptomycin
siRNA: Small interfering RNA
SEM: Scanning electron microscopy
RT-qPCR: Real-time quantitative polymerase chain reaction
NC: Negative control
ERK1/2: Extracellular-signal regulated kinase 1/2
JNK: c-Jun N-terminal kinase
p-p38: Phospho-p38
PDTC: Pyrrolidine dithiocarbamate
ER: Mineralocorticoid receptor
MDCK: Madin–Darby canine kidney
IL-1β: Interleukin-1β
