# Supplementary figures and images for: P38 MAPK signaling pathway mediates COM crystal-induced crystal adhesion change in rat renal tubular epithelial cells
Source: Urolithiasis. 2019 Jun 10;48(1):9–18. doi: 10.1007/s00240-019-01143-z (PMC6989645; doi:10.1007/s00240-019-01143-z)

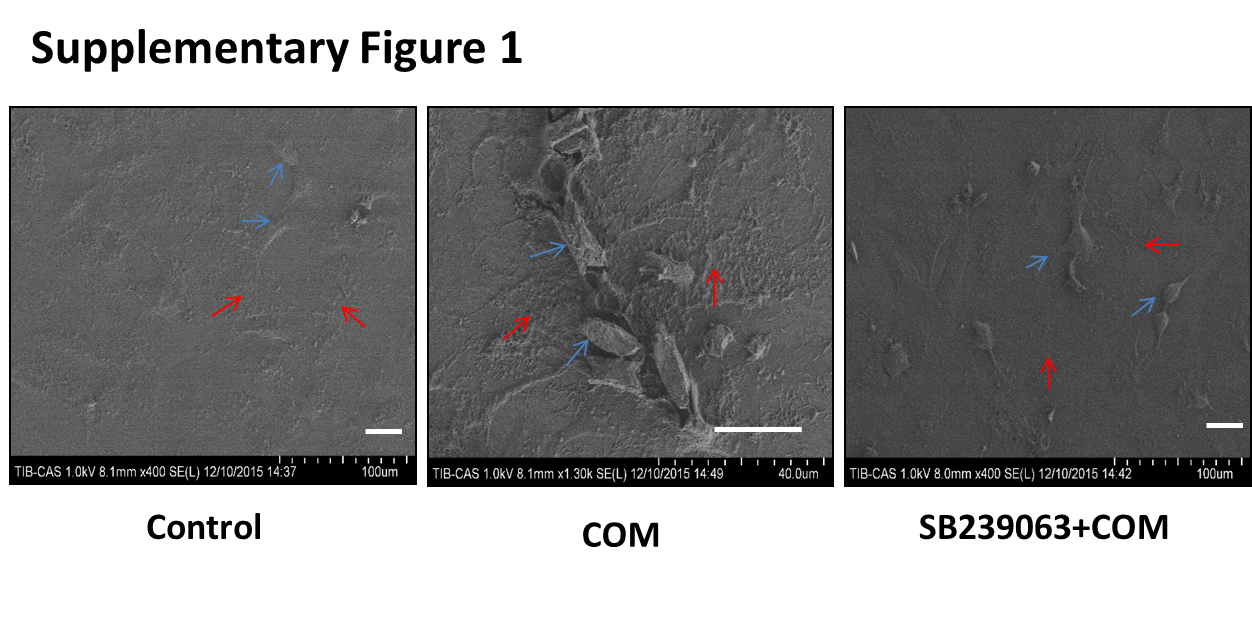

Supplement: Supplementary file 1 — Results of scanning electron microscopy. Controlled and COM-treated (146.0 µg/cm2) NRK-52E cells at 24 h, without or with 2-h pretreatment by SB239063 at 20 mM, were observed the changes of cell surface adhesion by SEM. COM crystal-treated cells had obvious swelling (blue arrows) and COM crystals adhesion significantly increased (red arrows). Scale bar is 20 µm [file 240_2019_1143_MOESM1_ESM.tif]
